# Unlocking Strength‐Toughness Dilemma in High‐Entropy Borides by Intragranular Microstructural Reconstruction

**DOI:** 10.1002/advs.76030

**Published:** 2026-06-28

**Authors:** Yingjun Liu, Yuhan Yao, Yufei Zu, Zhaofu Zhang, Yang Zhang, Hongfeng Dong, Nan Zhang, Wuhao Cao, Lehao Liu, Yuan Hu, Ruiheng An, Wenhu Li, Luyi Zhu, Taotao Ai

**Affiliations:** ^1^ School of Materials Science and Engineering Shaanxi University of Technology Hanzhong P. R. China; ^2^ National and Local Joint Engineering Laboratory For Slag Comprehensive Utilization and Environmental Technology School of Materials Science and Engineering Shaanxi University of Technology Hanzhong P. R. China; ^3^ School of Materials Science and Engineering Dalian University of Technology Dalian P. R. China; ^4^ Xi'an Aerospace Composites Research Institute Xi'an P. R. China; ^5^ State Key Laboratory of Crystal Materials Institute of Crystal Materials Shandong University Jinan P. R. China

**Keywords:** high‐entropy borides, interdiffusion, mechanical properties, non‐equilibrium sintering, strengthening and toughening

## Abstract

High‐entropy boride ceramics hold great promise as ultra‐high‐temperature structural materials but are hindered by the well‐known strength‐toughness trade‐off. Conventional extrinsic toughening approaches, such as composite reinforcement and microstructural refinement, offer limited improvements as they fail to modify the inherent intragranular tendency for brittle fracture. Here, we report an approach to overcome this limitation by constructing intragranular energy dissipation units through an extreme non‐equilibrium process. By employing heavy direct current sintering with TiSi_2_ addition, high densification (> 93% relative density) was achieved at a substantially reduced sintering temperature of 1000°C and an ultrahigh heating rate exceeding 5300°C/min. This process promotes selective diffusion of cations, leading to compositional redistribution within grains and forming compositional gradients and dislocation networks. These microscopic features collectively hinder crack propagation. The resulting ceramic demonstrates attractive mechanical properties with a flexural strength of 887 MPa and a fracture toughness of 7.1 MPa·m^1/2^. These findings demonstrate a viable pathway for the intrinsic toughening of high‐entropy ceramics through intragranular microstructural engineering.

## Graphical abstract

An extreme non‐equilibrium process combining heavy direct current sintering and TiSi_2_ addition is used to construct intragranular energy dissipation units within high‐entropy boride ceramics to overcome the strength‐toughness trade‐off. The resulting ceramic demonstrates attractive mechanical properties with a flexural strength of 887 MPa and a fracture toughness of 7.1 MPa·m^1/2^. This work provides a viable pathway for the toughening of brittle boride ceramics.

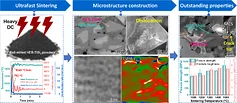

## 1 Introduction

Ultra‐high temperature ceramics (UHTCs), primarily consisting of refractory borides, carbides, and nitrides, form the cornerstone of thermal protection systems for hypersonic vehicles, next‐generation propulsion systems, and advanced nuclear reactors [1, 2]. Their unparalleled stability at temperatures exceeding 2000°C stems from strong covalent/ionic bonding. However, this same bonding characteristic is responsible for their fatal flaw: extreme brittleness and low damage tolerance, which persistently limit the reliability and lifespan of critical components [3].

The emergence of high‐entropy ceramics has infused new vitality into this field [4, 5, 6]. By incorporating multiple principal cations (typically five or more) in near‐equimolar ratios into a single‐sublattice crystal structure, these materials leverage high configurational entropy to stabilize simple solid solutions [7, 8]. High‐entropy borides (HEB) are a prominent class of UHTCs within this family [9, 10, 11]. The pronounced lattice distortion arising from the multi‐principal‐element cocktail can potentially enhance hardness, strength, and oxidation resistance, offering a promising avenue to surpass the performance limits of conventional binary borides [12, 13, 14, 15]. Consequently, HEBs are widely regarded as leading candidates for the next generation of ultra‐high‐temperature structural materials [3]. Despite this promise, the transition from laboratory curiosity to engineering application is fundamentally constrained, as the intrinsic brittleness of ceramics remains largely unaddressed by the high‐entropy design alone. Reported fracture toughness values for most bulk, single‐phase HEB, such as (TiZrNbHfTa)B_2_, consistently fall within a range of 2.5–4.5 MPa·m^1/2^ [16, 17, 18, 19]. This performance is comparable to, or even inferior to, that of their binary counterparts, revealing a critical bottleneck.

Research on toughening HEB has predominantly followed two extrinsic pathways. The first is composite toughening, which involves incorporating secondary phases like particles, whiskers, and fibers [20, 21]. These additives can deflect cracks and bridge crack surfaces, leading to measurable toughness improvements. For instance, the addition of SiC whiskers to a (TiZrNbHfTa)B_2_ composite was reported to increase fracture toughness to approximately 5.2 MPa·m^1/2^ [20]. The second relies on microstructural refinement via techniques like high‐energy ball milling and spark plasma sintering (SPS) to produce fine‐grained or nanocrystalline microstructures, which enhances hardness but offers limited gains in toughness [22, 23, 24, 25]. Critically, both strategies operate extrinsically: they introduce energy‐dissipation mechanisms outside the primary crystal lattice, such as at grain boundaries or phase interfaces, without modifying the intragranular propensity for brittle cleavage. This represents a fundamental physical limitation of the prevailing paradigm.

To address such challenges, researchers have begun exploring intrinsic toughening strategies that modulate the inherent fracture resistance of ceramics by controlling the composition, structure, and defects within the grains. Two prominent directions have emerged: phase inhomogeneity engineering and crystal defect engineering. Regarding phase inhomogeneity, Demirskyi et al. demonstrated that reactive spark plasma sintering of a ZrB_2_, tantalum, and amorphous boron powder mixture produced a hierarchical superstructure in Zr‐Ta multiborides, where the resulting (Zr,Ta)B_2_ solid solutions and composite architectures collectively improved mechanical properties [26]. Non‐equimolar (Zr_3_Ta_2_Nb)B_2_ ceramics were subsequently shown to exhibit a bending strength of 700 MPa at room temperature and 500 MPa at 1600°C, these values being approximately 1.7 times higher than for monolithic ZrB_2_ ceramics [27]. Non‑equimolar compositional design has also proven effective in carbide systems: Ta_0.2_Hf_0.8_C retains exceptional flexural strength up to 2000°C [28]. More recently, nanoscale phase inhomogeneity has been shown to govern mechanical performance in four‑cation high‑entropy diborides where grains are intrinsically heterogeneous at the nanoscale, comprising multiple complex diboride solid‑solution domains separated by compositionally graded interfaces [29]. This heterogeneity leads to remarkable high‐temperature ductility with a strain of approximately 7.5% and a flexural strength approximately 25% higher than the room temperature value. Beyond ceramic systems, sophisticated compositional design in complex metallic alloys has been shown to optimize property trade‐offs [30], as exemplified by TiAl complex alloys that achieve a gigapascal yield strength while retaining excellent ductility [31]. In parallel, crystal defect engineering has emerged as a powerful strategy, with recent demonstrations including the construction of ferrite‑domain/high‑density‑dislocation synergistic architectures in ferromagnetic oxide ceramics that increase fracture toughness by 100% [32], and the introduction of borrowed dislocations [33] and mechanically seeded dislocations [34] to significantly enhance the plastic deformation capacity of ceramics. While these advances collectively establish phase inhomogeneity and defect engineering as effective strategies for enhancing mechanical performance, their application to five‑cation high‑entropy borides remains largely unexplored.

In this work, we aim to explore the potential for enhancing the strength and toughness of five‑cation HEB by utilizing the intragranular compositional and defect heterogeneity. The (TiZrNbHfTa)B_2_ system was selected as the representative case, given that it is one of the most extensively investigated high‑entropy borides [19]. Owing to the intrinsic atomic size mismatch and chemical heterogeneity among its five constituent transition metals, this system develops pronounced lattice distortion, which is conducive to weakening B─B bonds and promoting dislocation nucleation [35]. To introduce intragranular heterogeneity into this matrix, TiSi_2_ was introduced as a reactive dopant to selectively modulate the diffusion behavior of various metallic cations [36, 37, 38]. Simultaneously, heavy direct current (DC) sintering was applied to drive the system far from equilibrium, with heating rates exceeding 10^3^°C/min and currents reaching several thousand amperes, thereby opening a kinetic window that suppresses complete homogenization [39, 40]. By combining an ultra‐rapid external physical field with an internally engineered chemical environment, intragranular reconstruction is induced, giving rise to core–shell compositional gradients and dislocation networks within HEB grains. The resulting HEB ceramic achieves an excellent balance of strength and toughness, with a flexural strength of 887 MPa and a fracture toughness of 7.1 MPa·m^1/2^. This study provides new insights into the intrinsic toughening of high‑entropy ceramics through deliberate intragranular microstructural engineering.

## 2 Results and Discussion

**FIGURE 1:**
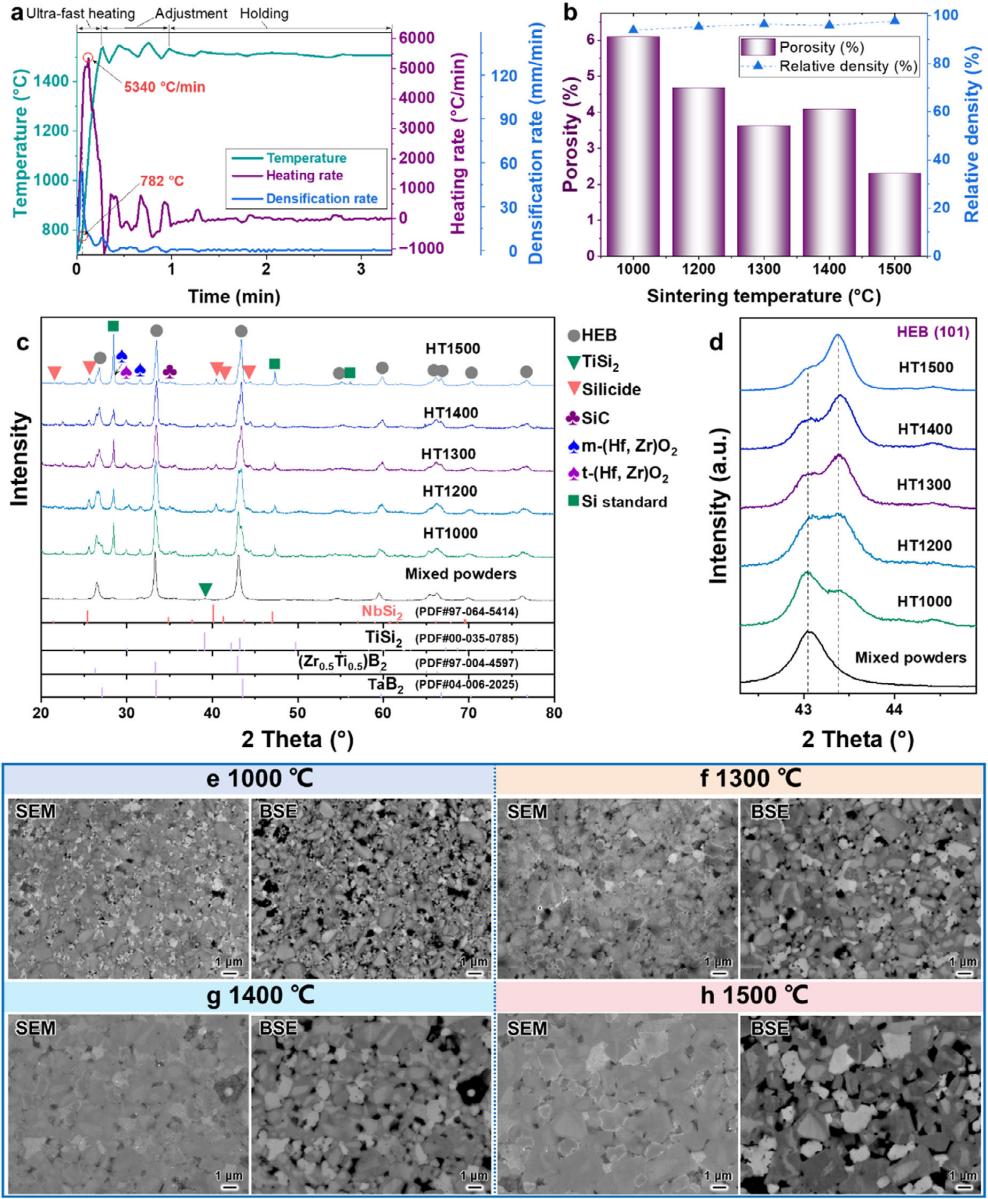
(a) Sintering curve of HEB‐TiSi_2_ ceramic via heavy  DC sintering, (b) porosity and relative density of HEB‐TiSi_2_ ceramics sintered at different temperatures; (c) XRD patterns of HEB‐TiSi_2_ ceramics, (d) enlarged pattern showing the (101) crystal plane diffraction peak of the HEB; SEM and BSE images of polished surface of HEB‐TiSi_2_ ceramics sintered at different temperatures: (e) 1000°C, (f) 1300°C, (g) 1400°C, (h) 1500°C.

To understand the densification behavior, shrinkage curves were recorded during the heavy direct current (DC) sintering process. Figure 1a presents the sintering curve for HEB‐based ceramic under a uniaxial 50 MPa load up to 1500°C. The differential displacement versus temperature curve illustrates the densification behavior, with the corresponding temperature differential curve provided for complementary analysis. As illustrated in Figure 1a, maximum shrinkage rate occurs at approximately 782°C. During the holding period, the shrinkage rate approaches zero, suggesting that near‐complete densification is achieved. A defining feature of this process is the extremely high heating rate, with a maximum recorded rate of 5340°C/min and an average rate of 3484°C/min. Such rapid heating rates significantly exceed those of conventional hot pressing or pressureless sintering (typically < 20°C/min) and even surpass many reported rates for spark plasma sintering of similar ultra‐high temperature ceramics (typically < 200°C/min), underscoring the extremely rapid heating capability of the heavy DC sintering process [41, 42]. This ultra‐fast sintering not only suggests the potential for shorter cycles and lower energy consumption but may also influence the dominant densification mechanisms by suppressing surface diffusion and enhancing bulk diffusion pathways [43]. Guided by the shrinkage profile in Figure 1a, a series of sintering experiments were conducted at 1000°C, 1200°C, 1300°C, and 1400°C. Porosity and relative density of the resulting samples are summarized in Figure 1b. A consistent and marked decrease in porosity is observed as the sintering temperature increases from 1000°C to 1500°C. The porosity drops from 6.1% at 1000°C to 2.3% at 1500°C, demonstrating the strong temperature dependence of the final density. In short, HEB was densified at a low temperature within a relatively short time, and the densification temperature was substantially lower than that typically reported for HEB‐based ceramics [10, 41].

X‐ray diffraction (XRD) analysis was conducted using Si powder as an internal standard to calibrate the peak positions. Results are summarized in Figure 1c,d. The XRD pattern of the as‐mixed powder (Figure 1c) confirms the presence of a single hexagonal solid‐solution HEB phase (space group P6/mmm), along with diffraction peaks corresponding to TiSi_2_ (PDF#00‐035‐0785). Following the ultrafast high‐temperature sintering cycle, TiSi_2_ diffraction peaks diminish and eventually disappear, while new peaks emerge at positions comparable to those of NbSi_2_, indicating a reaction or dissolution. Concurrently, a distinct peak splitting is observed within the major HEB phase. Figure 1d shows an enlarged view of the HEB (101) peak, where an additional peak appears at a higher diffraction angle. With increasing sintering temperature, the intensity of the original diffraction peak decreases, while that of the new high‐angle peak increases. This trend indicates a contraction of the unit cell, which is rationally attributed to the temperature‐activated, progressive diffusion of smaller Ti^4+^ cations into the HEB lattice, substituting for larger cations such as Nb^4+^ or Ta^4+^ and thereby inducing significant lattice strain [44].

Secondary electron images (SEM) and backscattered electron images (BSE) in Figure 1e–h illustrate the microstructural evolution of as‐sintered ceramics. The absence of significant pores indicates a high level of densification. Notably, distinct contrast variations within individual grains reveal compositional zoning characterized by a grayish‐white core surrounded by a gray rim, which forms a core–shell architecture. With increasing sintering temperature, both the thickness of the shell and the coarseness of the grains increase. The core–shell grains are uniformly dispersed throughout the matrix. Additionally, besides gray and grayish‐white phases, black and white phases were also observed, suggesting the formation of new species due to reactions between TiSi_2_ and HEB during sintering.

**FIGURE 2:**
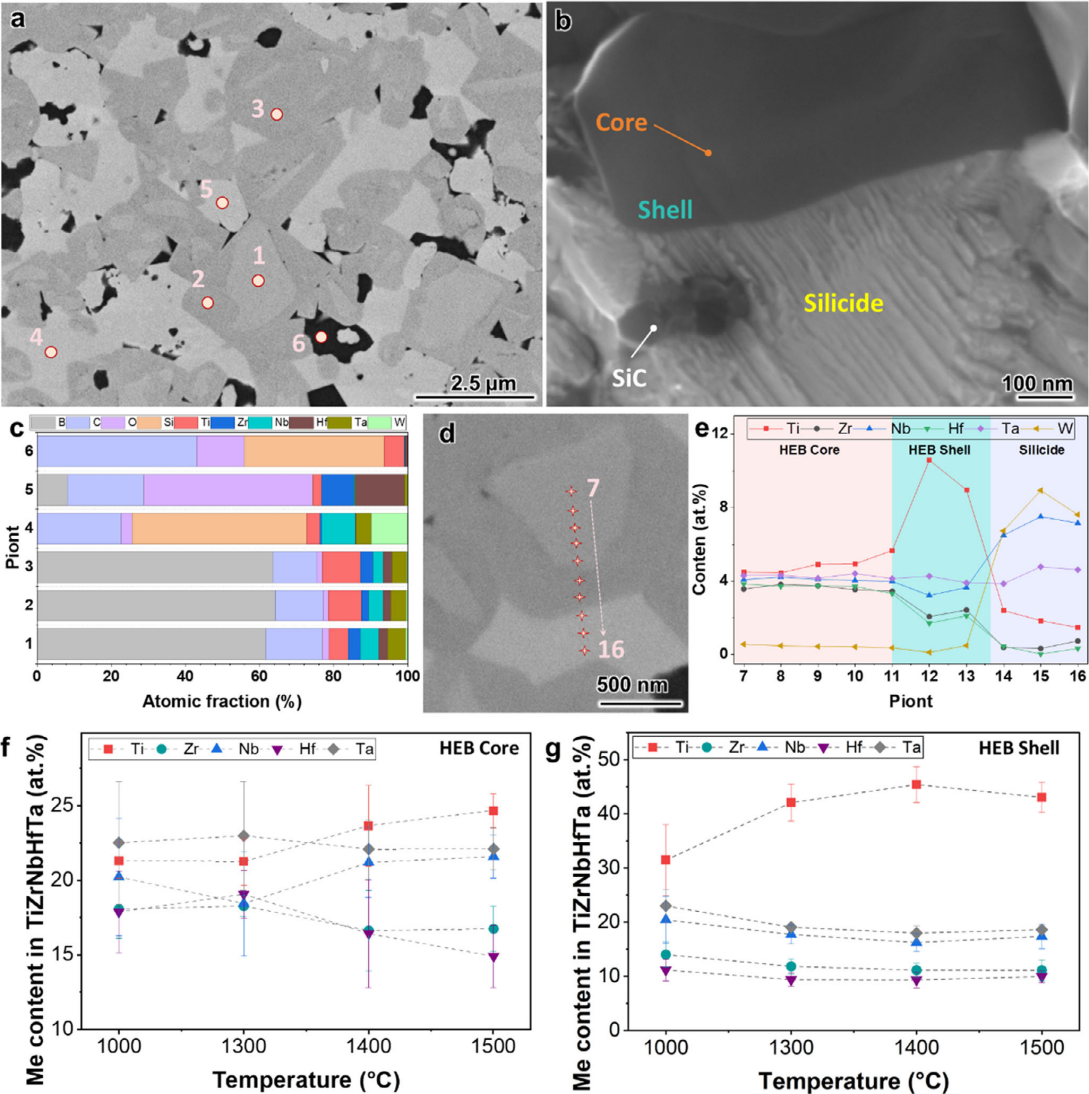
(a) BSE image of the HT1500, (b) SEM image revealing the layered structure of the silicide. (c) EDS spot analysis results of a select region marked in (a, d) continuous EDS spot analysis from borides to silicides, and (e) its results. Metal element content in high‐entropy borides: (f) HEB core and (g) HEB shell.

Figure 2 provides a comprehensive analysis of HT1500. In Figure 2a, a BSE image of HT1500 reveals four distinct phases, represented by white, grayish‐white, gray, and black contrast. Energy‐dispersive spectroscopy (EDS) spot analysis results of these phases are illustrated in Figure 2c. Figure 2d,e shows continuous EDS spot analysis of the HEB‐silicide region. From Figure 2a–c, it can be observed that the core–shell structure consists of a gray‐white HEB core encircled by a gray HEB shell, with the shell exhibiting higher Ti content. As shown in Table S1, the HEB core consistently retains a high‑entropy character (Δ*S_conf_* > 1.5 *R*) across all sintering temperatures. The HEB shell, by contrast, exhibits a systematic decrease in configurational entropy (Δ*S_conf_*) with increasing sintering temperature, transitioning from a marginal high‑entropy state at 1000°C (1.55 *R*) to a medium‑entropy state at 1300°C–1500°C (1.43–1.46 *R*). The black phase is likely SiC. Two types of white phase are identified: one with smooth grains corresponding to (Hf,Zr,Ti)O_2_, and another comprising a silicide containing Si, Nb, Ta, Ti, and W elements. The W element is possibly introduced during ball milling. Elemental profiles across the HEB core–shell region, as shown in Figure 2d,e, reveals a marked increase in Ti within the HEB shell, alongside elevated concentrations of Nb, Ta, and W toward the silicide. These results indicate that elemental interdiffusion occurred between TiSi_2_ and HEB during sintering, leading not only to the formation of core–shell HEB but also to the emergence of a new multi‐cationic silicide (MCS). The silicide phase consistently falls within the medium‑entropy regime across all sintering temperatures, with Δ*S_conf_* values ranging from 1.15 to 1.28 *R*. Finally, Figure 2b displays a high‐magnification SEM image of the HEB‐silicide interface, where the newly formed MCS exhibits a layered morphology.

**FIGURE 3:**
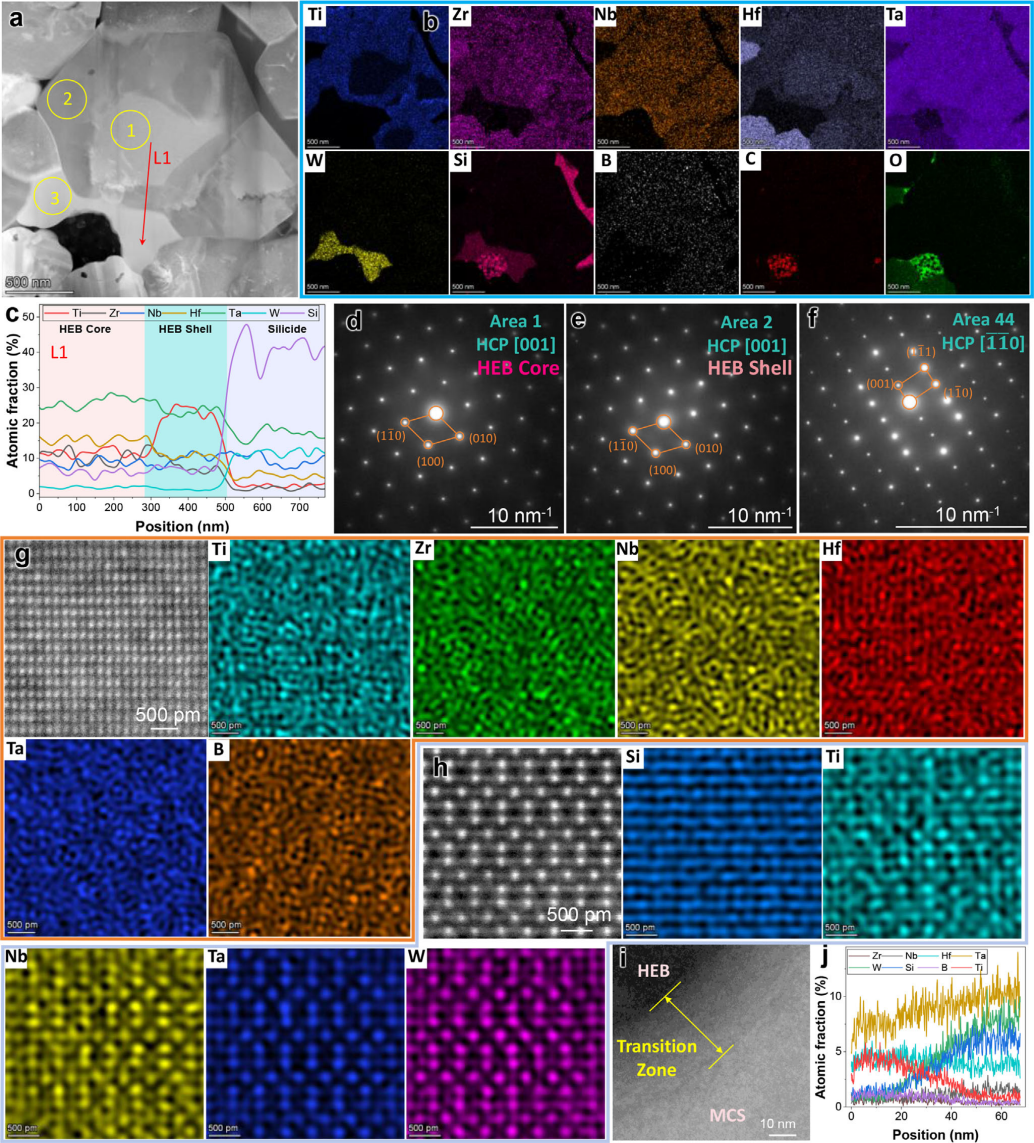
TEM analyses: (a) morphological images of HT1500 and (b) EDS mappings; (c) EDS line‐scan analysis of L1 marked in (a); (d–f) SAED pattern of area 1, 2, and 3 marked in (a); HAADF‐STEM image and the corresponding atomically resolved EDS mappings of (g) HEB and (h) MCS; (i) HRTEM of HEB‐MCS interface and (j) EDS line‐scan analysis of HEB‐MCS interface.

To identify the crystal structure and composition, transmission electron microscopy (TEM) was employed (Figure 3). Figure 3a,b presents a TEM image and the corresponding EDS element mappings, which distinguish between boride and silicide regions. Within the boride grains, Ti enrichment is evident near the shell, while in the silicide regions, Nb, Ta, and W are detected. An EDS line scan across the boride‐silicide interface (Figure 3c) further confirms Ti enrichment in the boride shell. Select area electron diffraction (SAED) patterns confirm that both the boride core and shell adopt a hexagonal structure (Figure 3d,e). Interestingly, the silicide diffraction pattern does not match orthorhombic TiSi_2_ (Figure 3f). Instead, it corresponds to the [$\bar{1}\bar{1}$0] zone axis of a hexagonal lattice with space group P622. EDS analyses reveal that the silicide phase contains Ti, Nb, Ta, and W in an atomic ratio of Ti:Nb:Ta:W = 0.11:0.35:0.17:0.37, with (Ti + Nb + Ta + W):Si = 1:1.78. Consequently, the nominal chemical formula of the silicide phase can be expressed as (Ti_0.11_Nb_0.35_Ta_0.17_W_0.37_)Si_1.78_. Taking into account both the atomic ratios and the selected area electron diffraction (SAED) pattern, the silicide phase is postulated to have the stoichiometry (Ti_0.11_Nb_0.35_Ta_0.17_W_0.37_)Si_2_. This phase is characterized by lattice parameters of a = 4.76 Å and c = 6.53 Å.

For further compositional elucidation, atomic‐resolution scanning transmission electron microscopy (STEM) and EDS mapping were conducted using a spherical‑aberration‑corrected TEM. Figure 3g,h shows the high‐angle annular dark‐field (HAADF)‐STEM image and corresponding EDS mappings of HEB and (Ti_0.11_Nb_0.35_Ta_0.17_W_0.37_)Si_2_ phases, respectively, revealing non‐uniform atomic‐level distributions of both transition‑metal and non‐metal elements in both phases. High‐resolution TEM (HRTEM) results reveal a transition zone approximately 30 nm wide between the HEB and MCS (Figure 3i). Elemental profiles across this interface (Figure 3j) indicate a gradual decrease in Ti intensity alongside increasing Ta, W, and Si signals within the transition region. This compositional gradient across the interface, rather than a sharp boundary, suggests the occurrence of active atomic interdiffusion during the sintering process, which may be driven by a reduction in interfacial energy between the two phases [45, 46].

To elucidate the temperature‐dependent compositional evolution of the core–shell HEB architecture, EDS analysis was conducted. For statistical reliability, at least ten representative areas were examined for each sample. The atomic proportion of each metal element (Me) relative to the total metal content was calculated, deliberately excluding boron to focus on the cation distribution. The results, summarized in Figure 2f,g, reveals a distinct compositional gradient between the core and shell across all samples. HEB shell consistently exhibits higher Ti content than the HEB core. As the temperature increased from 1000°C to 1400°C, the Ti content in the HEB shell increased from 31% to 45%, with a slight decrease observed at 1500°C. In contrast, the core demonstrated a more gradual Ti enrichment. The Ti content remained at comparable levels at 1000°C and 1200°C, followed by a steady increase to approximately 25% between 1300°C and 1500°C. These results indicate a temperature‐dependent evolution of the core–shell architecture. The concurrent thickening of the HEB shell with increasing temperature (Figure 1e–h) suggests a diffusion‐controlled growth mechanism. This phenomenon can be interpreted as a non‑equilibrium interdiffusion process driven by rapid sintering. During the initial sintering stage, Ti likely originating from decomposed or molten TiSi_2_ diffuses to the surface layer of HEB particles. The extremely short process duration characteristic of these methods kinetically hinders full homogenization, causing the structure to be frozen in a metastable state. This results in a Ti‐rich outer shell surrounding a Ti‐depleted core. Higher sintering temperatures provide greater kinetic energy, enhancing atomic mobility and driving the Ti‐rich diffusion front deeper into the grain, thereby increasing the shell thickness [37, 41]. Similar non‐homogeneous elemental distributions, where (Ta, Ti)‐rich and (Zr, Nb)‐rich regions coexist, have been observed in HEB powders synthesized at high temperatures, and complete homogenization often requires even higher temperatures or extended sintering times [47].

**FIGURE 4:**
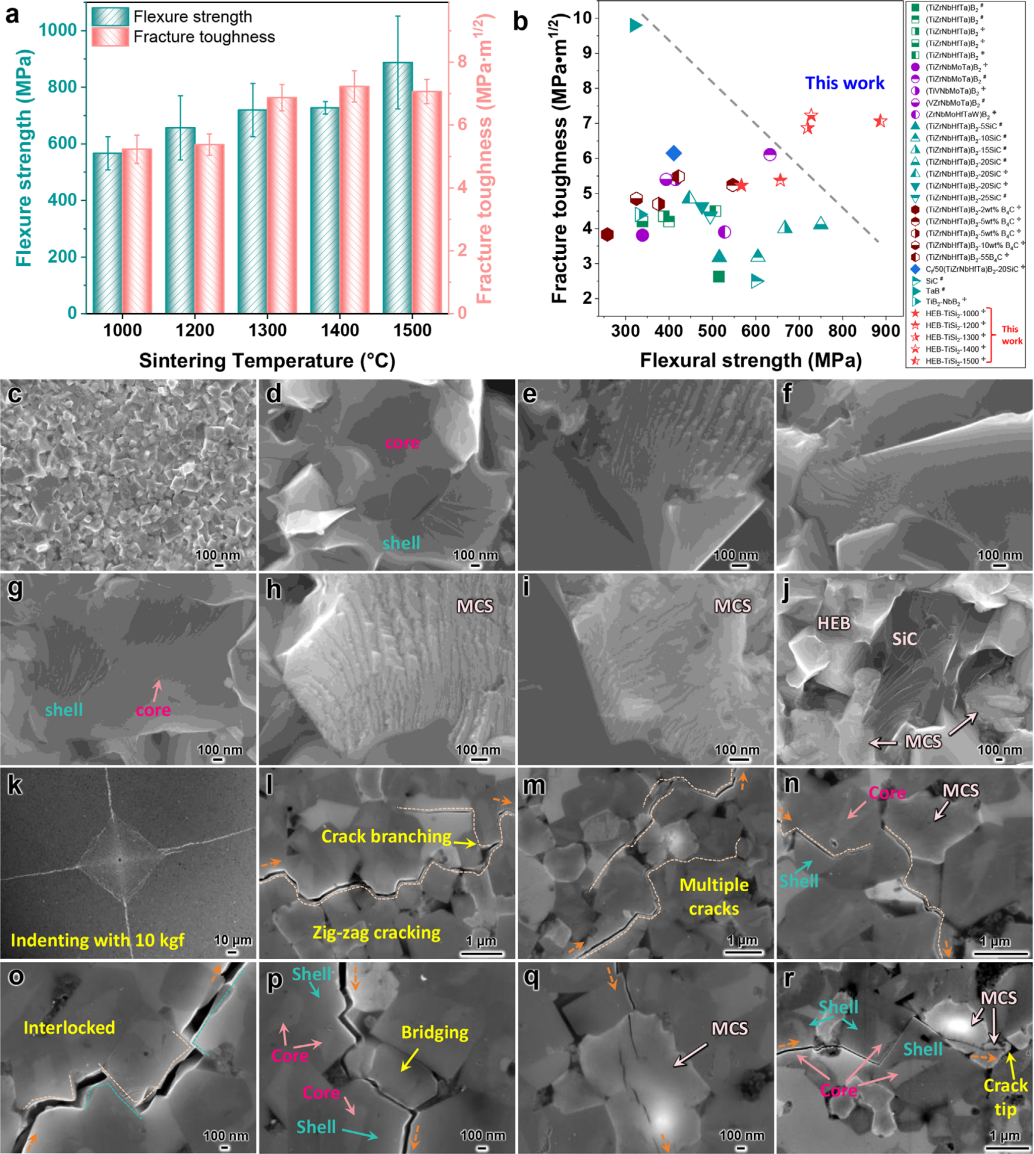
(a) flexural strength and fracture toughness of HEB‐TiSi_2_ ceramics sintered at 1000°C–1500°C, (b) Fracture toughness versus flexural strength plot comparing the HEB‐TiSi_2_ ceramics with other high‐entropy borides‐based composite [6, 10, 17, 18, 21, 22, 24, 48, 49, 50, 51, 52, 53, 54, 56]; The symbols #, 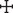, and 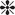 marked in (b) denote the indentation method, the single‐notched beam method, and the chevron notch beam method for determining fracture toughness, respectively; (c–j) SEM images of fracture surfaces of HZ1500, (k–r) SEM images of Vickers indentation produced cracks and crack propagation paths.

Flexural strength and fracture toughness of the HEB‐based ceramics as a function of sintering temperature are presented in Figure 4a. Flexural strength increases monotonically from 567 ± 58 MPa at 1000°C to 887 ± 164 MPa at 1500°C. Simultaneously, fracture toughness increases from 5.2 ± 0.5 MPa·m^1/2^ at 1000°C to a maximum of 7.2 ± 0.5 MPa·m^1/2^ at 1400°C, then slightly decreasing to 7.1 ± 0.4 MPa·m^1/2^ at 1500°C. Literature reports indicate that single‐phase HEB ceramics typically exhibit flexural strengths of 339–633 MPa and fracture toughness of 2.5–4.5 MPa·m^1/2^ [6, 10, 17, 18, 21, 22, 24, 48, 49, 50] (Figure 4b). The introduction of secondary phases, such as SiC or B_4_C, commonly enhances densification, inhibits grain growth, and refines microstructure, thereby improving flexural strength to approximately 750 MPa [18, 21, 24, 48, 51, 52]. Furthermore, incorporating reinforcing phases, such as carbon fibers, can further improve toughness through mechanisms like crack deflection, fiber bridging, and pull‐out, with reported fracture toughness reaching up to 6.15 MPa·m^1/2^ [21]. It is also noteworthy that Demirskyi et al. developed a TaB ceramic exhibiting a remarkably high room‑temperature fracture toughness of 9.8 MPa·m^1^/^2^, with flexural strength that continued to increase up to 1500°C [53]. In addition, polycrystalline SiC has been reported to retain a flexural strength as high as 2.08 GPa at 2000°C [54]. The present work demonstrates a superior combination of room‑temperature flexural strength and fracture toughness for HEB‑TiSi_2_ ceramic sintered at above 1200°C. Future studies will systematically evaluate the high‑temperature mechanical performance of these HEB‑TiSi_2_ ceramics and establish their position within the broader landscape of ultra‑high‑temperature structural materials.

Fracture surfaces of HZ1500 are shown in Figure 4c–j, revealing a mixed fracture mode of intergranular and transgranular characteristics. A distinct topographic contrast is evident between HEB core and HEB shell regions (Figure 4d–j). These HEB core regions appear either recessed or elevated relative to the surrounding HEB shell, resulting in a non‐planar, convoluted fracture topography across individual grains. This morphology suggests variations in mechanical properties or residual stresses between HEB core and HEB shell, likely arising from compositional gradients developed during sintering. The presence of a harder or more brittle phase may lead to fractures with limited deformation, generating height differences on the fracture plane [55]. Additionally, HEB shell regions occasionally display step‐like or terraced fracture patterns, which are characteristic of crack deflection and bifurcation at microstructural interfaces. When a propagating crack encounters an interface with weaker bonding or a mismatch in toughness (such as between HEB shell and adjacent phase), it may deviate, split, or be temporarily arrested. Such step‐wise crack propagation increases the total fracture surface area, thereby dissipating more fracture energy. In addition to the HEB matrix, the fracture behavior of secondary phases also plays a critical role. Both (Ti_0.11_Nb_0.35_Ta_0.17_W_0.37_)Si_2_ and SiC phases exhibit layered cleavage patterns (Figure 4i,j), indicative of a highly anisotropic, plate‐like crystal structure. Fracture in these phases occurs preferentially along weakly bonded crystallographic planes. The presence of such layered phases further contributes to toughening by promoting crack branching and microcracking ahead of the main crack tip, which effectively shields the crack and reduces the local stress intensity.

Crack propagation paths in the HEB‑based ceramics are illustrated in Figure 4k–r. Evidence of crack deflection, branching, and bridging is observed along the propagation trajectory (Figure 4l,m). Cracks propagate in a zigzag manner, exhibiting a non‐planar fracture mode. Particularly near core–shell HEB grains, cracks exhibit significant deflection, leading to complex propagation paths and the initiation of secondary cracks (Figure 4n–p). This behavior substantially increases energy dissipation during crack propagation, thereby enhancing fracture resistance. Moreover, crack branching occurs when cracks propagate through core–shell HEB grains and (Ti_0.11_Nb_0.35_Ta_0.17_W_0.37_)Si_2_ (Figure 4q,r). This phenomenon helps redistribute and weaken the localized driving force at the crack tip, thereby effectively inhibiting further crack propagation.

**FIGURE 5:**
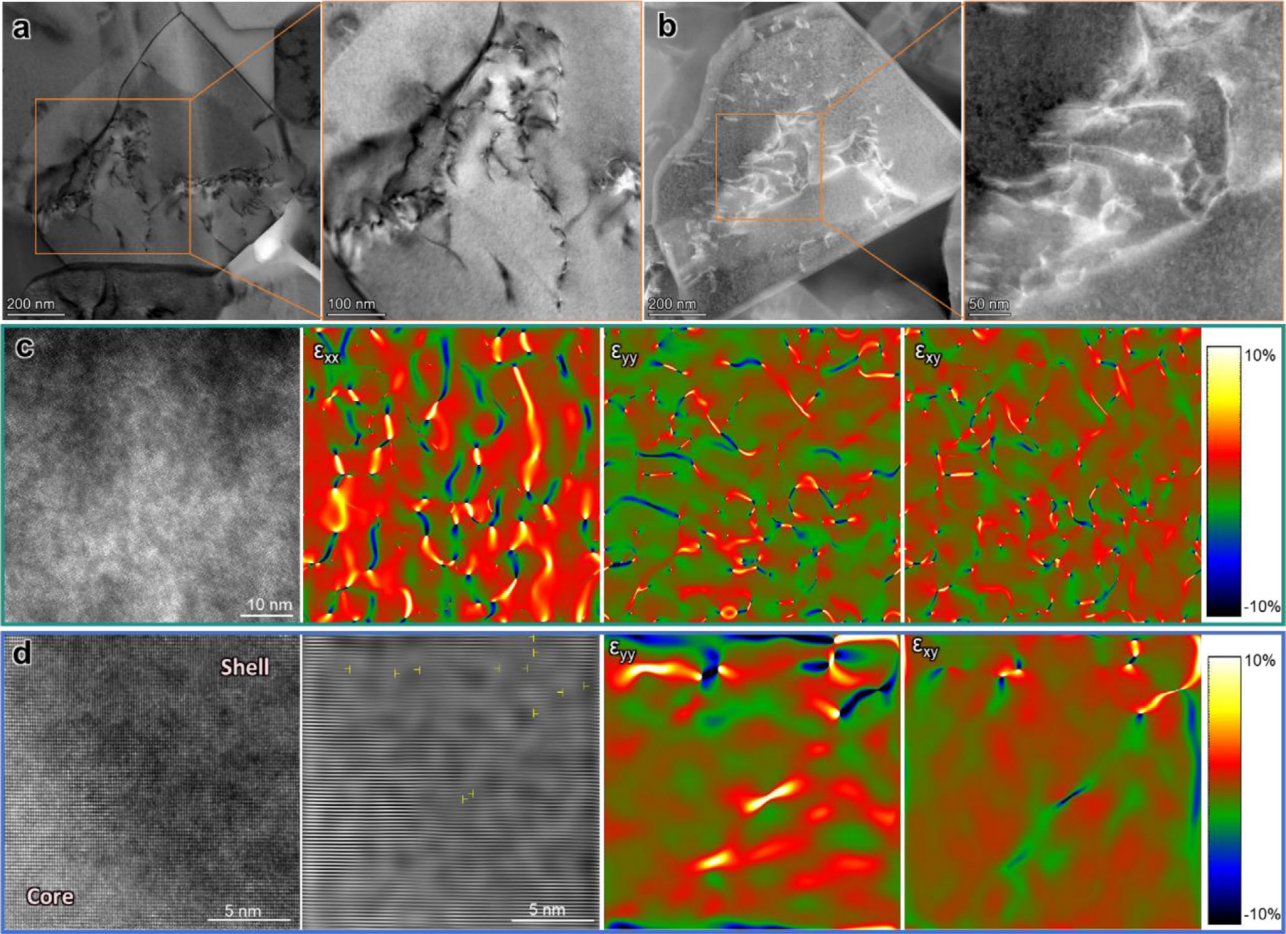
(a, b) TEM images of HEB‐TiSi_2_ ceramics, showing dislocations; HRTEM images and GPA analysis results from (c) high density of dislocations region and (d) the core–shell interfacial region.

To further elucidate the strengthening and toughening mechanisms, fine structures within the core–shell HEB were examined, and lattice strains were evaluated using geometric phase analysis (GPA). As shown in Figure 5a,b, dislocations are observed in both the HEB core and HEB shell. These dislocations likely originated from lattice distortions induced during processing and the mismatch in thermal expansion coefficients between the core and shell [57]. Such intentionally introduced or process‐inherited dislocations are now recognized as a viable microstructural tool to combat the inherent brittleness of ceramics by enhancing crack‐tip toughness [58]. The presence of dislocations can initiate shear‐coupled migration under stress, generating complex long‐range strain fields that significantly influence the material's mechanical response [45]. Using the GPA technique, in‐plane strain (ε_xx_), out‐of‐plane strain (ε_yy_), and shear strain (ε_xy_) distributions were obtained. Figure 5c illustrates the strain distribution in a dislocation‐rich region, where the strain field is clearly heterogeneous due to the influence of dislocations. Figure 5d presents GPA analysis results from the core–shell HEB interfacial region, showing that dislocations in the HEB shell induce a strongly non‐uniform strain field. This observed heterogeneity in the lattice strain is a direct consequence of the localized stress fields surrounding individual dislocations and their mutual interactions [45]. Reports indicated such microstructural features are conducive to hindering crack propagation by deflecting cracks and dissipating energy through interactions with the strain fields [58, 59]. Therefore, the interdiffusion‐driven formation of the core–shell structure in this work, along with the induction of dislocation formation and complex strain patterns, provides a viable approach for enhancing the mechanical properties of HEB‐based ceramic.

## 3 Conclusion

In summary, this work employed an extreme non‐equilibrium process that couples ultrafast heavy DC sintering (> 5300°C/min) with TiSi_2_ reactive doping to engineer intragranular energy‑dissipation units in (TiZrNbHfTa)B_2_‑based ceramics, effectively mitigating the long‑standing strength‐toughness trade‑off. The main findings are as follows:

1. The extreme non‐equilibrium process enables rapid densification of (TiZrNbHfTa)B_2_‐based ceramics at remarkably low temperatures, achieving > 93% relative density at just 1000°C and a final porosity of 2.3% at 1500°C.
2. Selective cation interdiffusion during the extreme non‐equilibrium process drives the formation of a core–shell boride grain architecture, consisting of a Ti‑depleted boride core and a Ti‑enriched boride shell, accompanied by the in‑situ formation of layered multi‐cationic silicides.
3. The intensified interdiffusion at elevated temperatures promotes non‑equilibrium compositional gradients and atomic‑scale heterogeneity, which in turn induce intragranular dislocation networks and non‑uniform lattice strain fields that serve as potent obstacles to crack propagation.
4. The (TiZrNbHfTa)B_2_‐based ceramics achieve an exceptional combination of flexural strength (887 MPa) and fracture toughness (7.1 MPa·m^1/2^), surpassing most previously reported HEB‑based ceramics.

These findings establish intragranular microstructural engineering via non‑equilibrium processing as a viable pathway for the intrinsic toughening of high‑entropy ultra‑high‑temperature ceramics.

## 4 Experimental Procedure

### 4.1 Powder Preparation and Sintering

(Ti_0.2_Zr_0.2_Nb_0.2_Hf_0.2_Ta_0.2_)B_2_ (HEB) powder was synthesized via a boron thermal reduction route. Commercially available oxide powders of TiO_2_, ZrO_2_, Nb_2_O_5_, HfO_2_, and Ta_2_O_5_ (all 99.9% purity, particle size 100 nm, Shanghai Chaowei Nano Technology Co., Ltd.) were used as metal precursors. These oxides were weighed in equimolar proportions corresponding to the target HEB stoichiometry and mixed with amorphous boron powder (99.9% purity, particle size 0.5–2 µm, Shanghai Macklin Biochemical Co., Ltd.). To compensate for boron loss during the subsequent high‐temperature synthesis, an excess of 17 mol% boron relative to the stoichiometric requirement was introduced. The powder mixture was ball‐milled in ethanol for 12 h. After drying, the mixed powder was subjected to a two‐stage heat treatment: it was heated to 1700°C and held for 2 h under vacuum at 1700°C to complete the boro‐thermal reduction and solid‐solution formation, followed by an additional 0.5‐h hold under vacuum at 1700°C to remove volatile byproducts, yielding the single‐phase HEB powder. Commercial TiSi_2_ powder (purity > 99%, particle size 2–5 µm, Shanghai Macklin Biochemical Co., Ltd) served as the reactive additive. The as‐synthesized HEB powder and commercial TiSi_2_ powder were blended to a composition of 80 vol% HEB and 20 vol% TiSi_2_. The mixture was ball‐milled for 36 h. The slurry was then dried in a vacuum and sieved.

Consolidation of the HEB‐TiSi_2_ powder was conducted using a fast hot‐pressed sintering system (FHP828, Suzhou Hateng Technology Co., Ltd.). Unlike spark plasma sintering (SPS) systems that utilize a pulsed direct current (DC) power supply, this system is equipped with a continuous DC power supply. The sintered samples were produced with final dimensions of 20 × 20 × 6 mm^3^. To enhance heating efficiency, an improved multilayer mold was employed, as detailed in our previous work [60]. A schematic diagram of the experimental setup is provided in Figure S1. Briefly, the multilayer mold consisted of an inner graphite liner, a boron nitride (BN) insulating interlayer, and an external graphite mold. The entire sintering process was conducted under a vacuum. A uniaxial pressure of 50 MPa was applied and maintained throughout the sintering cycle. Sample temperature was monitored using a two‐color infrared thermometer (STRONG‐SR‐7025WL, Changzhou Sijie Optoelectronics Technology Co. Ltd., China), with the measurement point positioned approximately 2 mm from the sample. During the heating process, a constant DC of 5000 A was utilized for rapid heating. As the sample temperature approached the target sintering temperature (within approximately 50°C below the setpoint), the operator manually adjusted the applied DC current based on real‐time feedback from the infrared thermometer. The total duration of this regulated period was 3 min, encompassing both the initial stabilization (adjustment stage) and the subsequent isothermal dwell (holding stage). According to the binary Ti‐Si phase diagram, TiSi_2_ melts at approximately 1488°C [61]. Sintering near this temperature is expected to promote the formation of transient liquid phases, thereby promoting elemental interdiffusion and densification. Therefore, HEB‐TiSi_2_ was sintered at 1500°C and labeled as HT1500. Real‐time displacement monitoring, calibrated against blank reference samples, was employed to analyze densification behavior. Based on the shrinkage curve of the HT1500 sample, HEB‐TiSi_2_ was sintered at 1000°C, 1200°C, 1300°C, and 1400°C, labeled as HT1000, HT1200, HT1300, and HT1400, respectively. All sintering runs used identical molds and nominally equivalent powder.

### 4.2 Characterization

Bulk density of the sintered samples was determined by the Archimedes method. Theoretical density was calculated via a rule of mixtures, adopting densities of 8.34 g/cm^3^ for HEB and 4.39 g/cm^3^ for TiSi_2_. Relative density and porosity were derived accordingly.

Phase identification was performed via X‐ray diffraction (XRD, Rigaku SmartLab) over a 2θ range of 20°–80° with a step size of 0.01° and a scanning speed of 1°/min. Microstructural characterization was carried out using scanning electron microscopy (SEM, JEOL 7610F Plus) equipped with a back‐scattered electron (BSE) detector. Elemental analysis was conducted using energy‐dispersive X‐ray spectroscopy (EDS, Ultim Extreme). A field‐emission transmission electron microscope (Talos F200XG2) and a spherical aberration correction transmission electron microscope (Titan Cubed Themis G2 300) with selected‐area electron diffraction (SAED) and EDS were employed for nanoscale structural and chemical analysis. The samples for transmission electron microscopy (TEM) observation were prepared using a focused ion beam (TESCAN AMBER GMH).

Flexural strength was evaluated via a three‐point bending test using a universal testing machine (WDW‐20S, Shandong Lisu Testing Equipment Co., Ltd., China) on bars with a cross‐section of 2 mm × 2.5 mm and a span of 18 mm, at a crosshead speed of 0.5 mm/min. Fracture toughness was assessed via the single‐edge notched beam (SENB) method on the same testing machine using bars with a cross‐section of 4 mm × 2 mm, with a notch depth of 2 mm and a width of less than 0.3 mm. All mechanical tests were conducted in ambient air at room temperature. At least three specimens were tested under each condition. To examine the crack extension behavior, selected samples were indented under a 10 kgf load, and crack propagation paths were analyzed using SEM.

## Author Contributions

**Yingjun Liu**: conceptualization, investigation, formal analysis, funding acquisition, writing – original draft, writing – review and editing. **Yuhan Yao**: investigation, formal analysis, writing – original draft, writing – review and editing. **Yufei Zu**: formal analysis, writing – review and editing. **Zhaofu Zhang**: formal analysis, writing – review and editing. **Yang Zhang**: formal analysis, funding acquisition, writing – review and editing. **Hongfeng Dong**: funding acquisition, formal analysis, writing – review and editing. **Nan Zhang**: formal analysis, funding acquisition, writing – review and editing. **Wuhao Cao**: formal analysis, writing – review and editing. **Lehao Liu**: formal analysis, writing – review and editing. **Yuan Hu**: formal analysis, writing – review and editing. **Ruiheng An**: formal analysis, writing – review and editing. **Wenhu Li**: formal analysis, writing – review and editing. **Luyi Zhu**: formal analysis, funding acquisition, writing – review and editing. **Taotao Ai**: conceptualization, formal analysis, writing – review and editing.

## Funding

This work was supported by National Natural Science Foundation of China (Grant No. 52572320); Natural Science Basic Research Program of Shaanxi (Grant Nos. 2024JC‐YBQN‐0580, and 2025JC‐YBMS‐403); Key Research and Development Program of Shaanxi (Grant No. 2025JC‐QYCX‐042); Scientific Research Program Funded by Education Department of Shaanxi Provincial Government (Grant Nos. 25JP031, and 25JK0379); Science Foundation of Shaanxi University of Technology for Youths (Grant No. SLGRCQD015); and Open Research Projects of the Shaanxi Provincial Key Laboratory of Advanced Manufacturing and Health Management for Aviation Components (Grant No. SLGKFKT06).

## Conflicts of Interest

The authors declare no conflicts of interest.

## Supporting information




**Supporting File**: advs76030‐sup‐0001‐SuppMat.docx.

## Data Availability

The data that support the findings of this study are available from the corresponding author upon reasonable request.
