# Supplementary material for: Unlocking Strength‐Toughness Dilemma in High‐Entropy Borides by Intragranular Microstructural Reconstruction
Source: Adv Sci (Weinh). 2026 Jun 28:e76030. Online ahead of print. doi: 10.1002/advs.76030 (PMC13336881; doi:10.1002/advs.76030)
Supplement: Supplementary file 1 — Supporting File: advs76030‐sup‐0001‐SuppMat.docx. [file ADVS-9999-e76030-s001.docx]

Supporting Information

Unlocking Strength-Toughness Dilemma in High-Entropy Borides by Intragranular Microstructural Reconstruction

Yingjun Liu^a, b^, Yuhan Yao^a^, Yufei Zu^c^, Zhaofu Zhang^d^, Yang Zhang^a, b^, Hongfeng Dong^a, b^, Nan Zhang^a, b^, Wuhao Cao^a^, Lehao Liu^a^, Yuan Hu^a^, Ruiheng An^a^, Wenhu Li^a, b^, Luyi Zhu^e^, Taotao Ai^a, b, *^

^a^ School of Materials Science and Engineering, Shaanxi University of Technology, Hanzhong, 723001, P.R. China.

^b^ National and Local Joint Engineering Laboratory for Slag Comprehensive Utilization and Environmental Technology, School of Materials Science and Engineering, Shaanxi University of Technology, Hanzhong, 723001, P.R. China.

^c^ School of Materials Science and Engineering, Dalian University of Technology, Dalian, 116024, P.R. China.

^d^ Xi'an Aerospace Composites Research Institute, Xi'an, 710025, P.R. China.

^e^ State Key Laboratory of Crystal Materials, Institute of Crystal Materials, Shandong University, Jinan 250100, P.R. China.

* Corresponding author.

aitaotao0116@126.com (Taotao Ai)


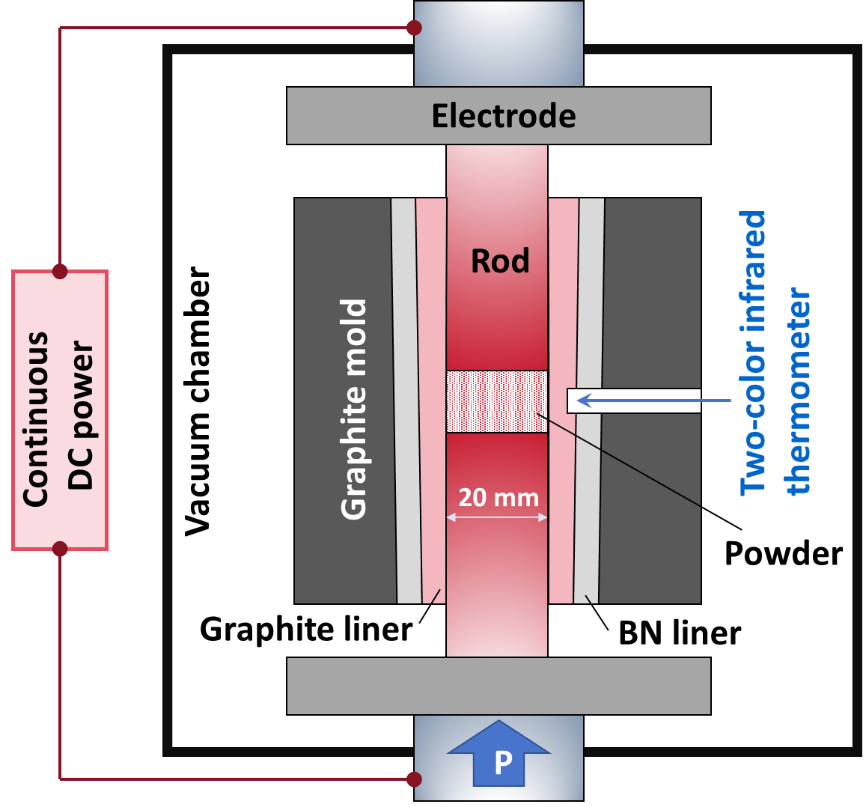


**Figure S1.** Scheme diagram of sintering equipment.

Configurational entropy was estimated using the following equation [1]:

|  | $\Delta S_{conf}=-R\sum_{i=1}^{n} x_{i}lnx_{i}$ | (S1) |
| --- | --- | --- |

Where *R* is the gas constant and $x_{i}$ is the atom fraction of element *i*. The calculation results for $\Delta S_{conf}$ of HEB core, HEB shell and silicide are summarized in Table S1.

**Table S1.** Configurational entropy of HEB core, HEB shell and silicide.

|  | HEB core | HEB shell | silicide |
| --- | --- | --- | --- |
| HT1000 | 1.61 R | 1.55 R | 1.27 R |
| HT1300 | 1.61 R | 1.46 R | 1.15 R |
| HT1400 | 1.60 R | 1.43 R | 1.22 R |
| HT1500 | 1.59 R | 1.45 R | 1.28 R |

**References**

1. D. B. Miracle, O. N. Senkov,"A critical review of high entropy alloys and related concepts," *Acta Materialia* 122, (2017): 448. <https://doi.org/10.1016/j.actamat.2016.08.081>.
